# Participant-Aware Model Validation for Repeated-Measures Data: Comparative Cross-Validation Study

**DOI:** 10.2196/87728

**Published:** 2026-04-30

**Authors:** Abdolamir Karbalaie, Farhad Abtahi, Charlotte K Häger

**Affiliations:** 1 Department of Community Medicine and Rehabilitation Umeå University Umeå, Västerbotten Sweden; 2 Department of Biomedical Engineering and Health Systems, School of Engineering Sciences in Chemistry, Biotechnology and Health KTH Royal Institute of Technology Huddinge, Stockholm Sweden; 3 Department of Clinical Science, Intervention and Technology Karolinska Institutet Huddinge, Stockholm Sweden; 4 Department of Clinical Physiology Karolinska University Hospital Stockholm Sweden

**Keywords:** data leakage prevention, machine learning validation, human movement control, model selection bias, cross-validation benchmarking, transparent AI evaluation

## Abstract

**Background:**

Repeated-measures datasets are common in biomechanics and digital health, where each participant contributes multiple correlated trials. If cross-validation (CV) ignores this structure, information can leak from training to test folds, inflating performance and undermining clinical credibility.

**Objective:**

This study evaluates the impact of participant-aware validation strategies on model reliability in repeated-measures classification tasks, using fear of reinjury prediction following anterior cruciate ligament reconstruction (ACLR) as a case study.

**Methods:**

We analyzed 623 hop trials from 72 individuals after ACLR to classify fear of reinjury based on biomechanical features. Four CV strategies were compared: stratified 10-fold CV, leave-one-participant-out cross-validation (LOPOCV), group 3-fold CV, and a nested framework combining LOPOCV (outer loop) with group 3-fold CV (inner loop). Ten supervised classifiers were benchmarked across classification accuracy, train-test generalization gap, model ranking consistency, and computational efficiency.

**Results:**

Stratified 10-fold CV systematically overestimated model performance (eg, extra trees accuracy of 0.91 vs 0.66 under LOPOCV) due to participant-level data leakage. Group and nested CV strategies yielded more conservative and stable estimates. The nested LOPOCV + group CV framework achieved a good balance between generalization and participant-aware separation, with reduced bias and overfitting compared with nonnested alternatives.

**Conclusions:**

Participant-aware validation strategies are essential for trustworthy machine learning (ML) evaluation in repeated-measures settings. Nested CV designs improve reproducibility, reduce selection bias, and align with regulatory expectations for clinical ML tools. These findings support best practices in model validation for biomechanics and digital health applications.

## Introduction

Machine learning (ML) is becoming increasingly central to analyzing complex human data gathered through repeated trials, particularly in biomechanics, clinical assessment, and digital health contexts. These datasets, often comprising multiple sessions or sensor recordings per participant, provide insight into within-person variability and strengthen longitudinal evaluations. In biomechanics, repeated gait and hop assessments quantify within-person variability and recovery [1,2]. Similar repeated designs track performance shifts in sports contexts [3].

In clinical and behavioral research, accounting for repeated measures is crucial for making valid inferences. Powell et al [4] applied repeated-measures analysis of covariance to assess how breast support influences running biomechanics while controlling for within-participant variation. Similarly, Keogh et al [5] used repeated-measures correlation to separate individual-level patterns from group-level effects in a cohort of athletes. These examples highlight the necessity of modeling within-participant structures to draw meaningful conclusions.

With the rise of wearable technologies and sensor-based monitoring, repeated-measures data have become increasingly prevalent in applications ranging from cardiac diagnostics to musculoskeletal load tracking [6,7]. When ML evaluation ignores participant identity, trial-level dependencies can leak across folds, inflating performance and undermining generalizability [8-10]. Despite broad awareness of this risk, stratified K-fold CV is still commonly used in ways that overlook subject boundaries. This creates data leakage, as correlated trials from the same participant can appear in both training and test sets. Studies in neuroimaging, ophthalmology, and sensor-based health applications have reported this pattern [11-13]. Our companion standardized rebound side hops (SRSH) study addressed the same clinical construct using the entire biomechanical time series with a 1D convolutional neural network under leave-one-participant-out cross-validation (LOPOCV) [14]. That analysis demonstrated that participant-wise evaluation is crucial for credible accuracy on repeated hop trials. That study held model hyperparameters fixed across folds; here, we systematize the validation question by comparing participant-aware CV designs and, when tuning is required, separating it from evaluation.

Participant-aware validation strategies such as LOPOCV and group K-fold CV have been proposed to enforce participant-level separation during evaluation [15,16]. However, these strategies differ in how they handle intraparticipant variation during hyperparameter tuning, and simple (nonnested) implementations can still introduce selection bias. Nested CV frameworks, which decouple tuning from evaluation, offer a principled alternative to reduce selection bias and overfitting, but adoption remains limited in clinical and behavioral ML [8]. Crucially, the field lacks a systematic benchmark that goes beyond accuracy to quantify (1) validation bias, (2) train-test gaps, (3) participant-level rank stability, and (4) computational efficiency across commonly used models—limitations that constrain reproducibility and practical deployment.

This study addresses this need by providing a systematic, multidimensional benchmark of cross-validation (CV) strategies for repeated-measures clinical ML data. We compare 4 CV designs, including a fully nested framework with LOPOCV for outer-loop evaluation and group K-fold (K=3) for inner-loop model selection, across 10 classifiers and the 4 evaluation axes listed above. The anterior cruciate ligament reconstruction (ACLR) fear-of-reinjury classification task serves as a representative case study; the primary contribution is the validation methodology, which generalizes to other repeated-measures clinical settings. The study follows the TRIPOD+AI (Transparent Reporting of a multivariable prediction model for Individual Prognosis Or Diagnosis—Artificial Intelligence extension) guidelines [17], and the completed checklist is provided in Table S1 in Multimedia Appendix 1. The following research questions were posed to guide the investigation:

- Does standalone LOPOCV overestimate performance by neglecting within-participant variation during tuning?
- Can group K-fold (K=3) CV improve model selection by leveraging repeated trials?
- Does a nested LOPOCV (outer) + group K-fold CV (inner) strategy yield more stable and realistic performance estimates for repeated-measures classification?

## Methods

### Study Cohort

This study included 72 individuals (43 males and 29 females; age: mean 28.4 years, SD 6.7 years), all between 6 and 24 months after unilateral ACLR using a hamstring autograft.

### Ethics Considerations

This study was approved by the Regional Ethical Review Board in Umeå, Sweden (approval numbers 2015/67-31 and 2021-03860). All participants provided written informed consent before participation. Participant data were deidentified before analysis and handled in accordance with applicable Swedish regulations on privacy, confidentiality, and patient safety. Participants did not receive any compensation.

### Biomechanical Data Collection

We used the SRSH test to evaluate lateral dynamic stability, neuromuscular control, and lower-limb coordination under sport-specific plyometric loading [18]. During each trial, participants performed single-leg lateral hops across 2 adjacent force plates, with an emphasis on controlled landings and immediate rebounds.

Each participant completed between 5 and 10 valid SRSH trials under standardized protocol conditions. Three-dimensional motion capture was conducted using an 8-camera optical system (Qualisys Oqus 300, 240 Hz), synchronized with dual force platforms (Kistler 9286AA, 1200 Hz). A 56-marker full-body setup captured segmental joint motion.

### Data Preprocessing

Marker trajectories were processed in Qualisys Track Manager (version 2019.3) using a zero-lag, fourth-order low-pass Butterworth filter (12 Hz for markers; 50 Hz for force data). The filtered data were exported to Visual3D (C-Motion Inc) for calculation of joint angles and moments via inverse dynamics. All kinetic variables were normalized to body mass.

The stance phase—defined from initial ground contact to the lowest vertical position of the center of mass—was segmented and time-normalized to 101 points using third-order polynomial fitting. Trial-level metadata, including participant identifiers *p_i_* and trial indices *t_i_*, were retained for participant-aware modeling and validation.

### Target Labeling: Fear of Reinjury

After preprocessing, fear of reinjury was assessed using item 9 of the 17-item Tampa Scale of Kinesiophobia, which states “I am afraid that I might injure myself accidentally.” Following the classification procedure proposed by Markström et al [19], participants who responded “agree” or “strongly agree” were categorized as having high fear (n=36 participants, 301 trials), and those who responded “disagree” or “strongly disagree” were categorized as having low fear (n=36 participants, 322 trials). These binary categories served as target labels for the classification task.

### Feature Engineering and Selection

Time-series descriptors were generated with TSFRESH [20] from 48 biomechanical signals (angles and moments; lateral/medial landings). We retained 13 summary statistics per signal (mean, SD, variance, skewness, kurtosis, 10th/50th/90th percentiles, autocorrelation at lags 1 and 2, counts above/below the mean, and absolute sum of changes), yielding 624 features per trial.

TSFRESH’s built-in hypothesis tests were applied at α=.05 with false-discovery control to remove noninformative descriptors. Surviving features were ranked by Gradient Boosting Classifier (GBC) importance, computed on the pooled training data used for feature preparation. The ranking produced top-k subsets (top 5-10 [step size 1], then 20-100). To keep inputs compact, stable, and fast to evaluate—and to avoid mixing feature selection with performance reporting—we fixed k=10 a priori and used the corresponding top-10 set for all CV experiments. This choice reflects diminishing returns beyond small k on our development trials and keeps the focus on the validation design rather than on feature-count tuning. Importantly, the top-10 feature set was determined before any CV experiment began and was held constant across all folds and strategies. The features were selected using domain-relevant biomechanical reasoning and a single, preexperimental GBC ranking, not through a data-driven search repeated within each fold. As a result, no information from test folds influenced the feature set, eliminating feature-selection bias from the validation comparison. Correlated features (eg, bilateral joint angles) were intentionally retained, as the goal was to evaluate validation-strategy effects under a fixed, realistic feature set rather than to optimize feature independence.

**Figure 1:**
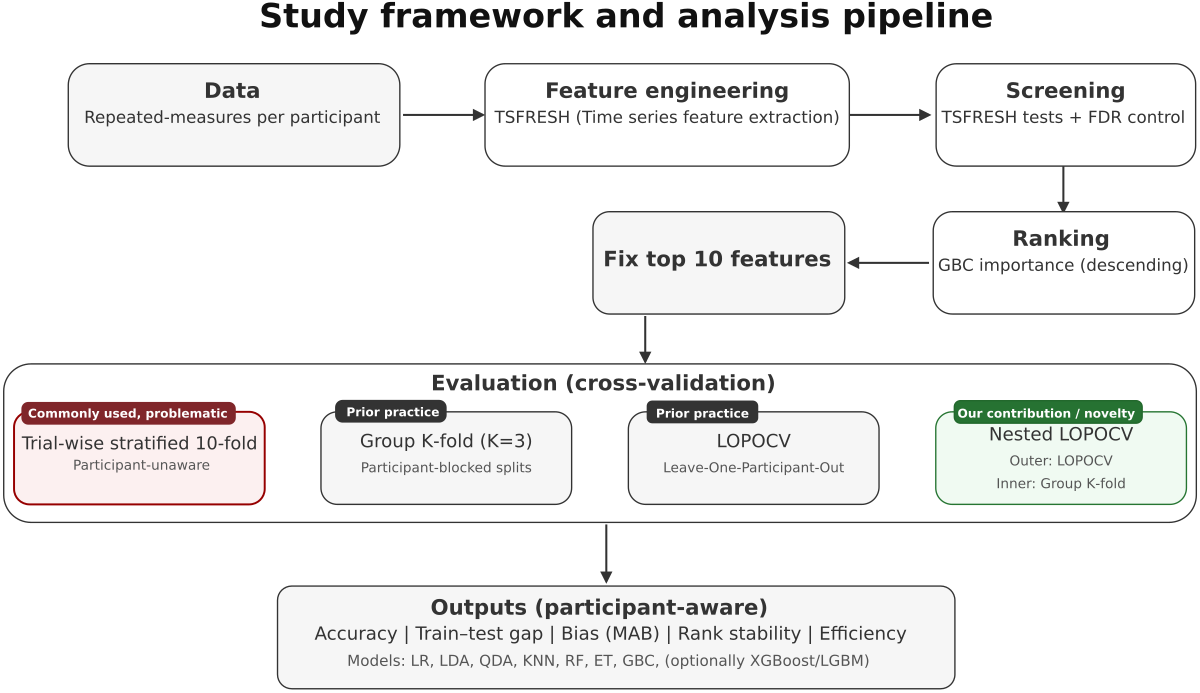
Study framework for participant-aware validation. Raw kinematic and kinetic time-series data are first summarized using TSFRESH feature extraction. Features are then screened with statistical tests applying false discovery rate (FDR) correction, ranked using a Gradient Boosting Classifier (GBC), and selected at multiple thresholds for subsequent model training and evaluation. Panel labels indicate category (contribution, prior practice, commonly used, problematic). Colors are supplemented with text labels to ensure readability. ET: extra trees; KNN: k-nearest neighbors; LDA: linear discriminant analysis; LOPOCV: leave-one-out cross-validation; LR: logistic regression; QDA: quadratic discriminant analysis; RF: random forest.

The top-10 ranked features (with signal/statistic provenance) are listed in Table 1 and were held fixed across all models and validation schemes. Within each fold, these features were z-scaled (using StandardScaler from scikit-learn) on the training split and then applied to the corresponding test split. Feature ranks are documented to specify model inputs for the validation comparison. They are not advanced as biomarker evidence; this study aimed to examine participant-aware evaluation and performance inflation when participant identity is ignored. An overview of this feature pipeline and its integration with the evaluation design is shown in Figure 1.

**Table 1:** Summary of the top 10 selected features for classifying fear of reinjury^a^.

Joint	Plane	Side	Statistic
Ankle	Supination/pronation	Medial	Absolute sum of changes
Hip	Rotation	Medial	Mean
Ankle	Rotation	Medial	90th percentile
Trunk	Lean (lateral flexion)	Lateral	Kurtosis
Ankle	Flexion/extension	Lateral	10th percentile
Knee	Rotation	Lateral	Skewness
Hip moment	Rotation	Medial	Absolute sum of changes
Knee	Rotation	Medial	10th percentile
Hip	Adduction/abduction	Lateral	Median (50th percentile)
Knee	Adduction/abduction	Medial	Median (50th percentile)

^a^Joint-level sources, anatomical planes, body side, and statistical descriptors were extracted from side hop rebound trials. Features were selected based on the Gradient Boosting Classifier importance ranking after univariate filtering.

### Machine Learning Algorithms and Hyperparameter Tuning

#### Classifiers

Ten supervised classification models were selected based on their frequent use in biomechanical and clinical ML tasks, representing a range of algorithmic families. These included 3 linear models—logistic regression (LR), linear discriminant analysis (LDA), and quadratic discriminant analysis (QDA); 1 instance-based method—k-nearest neighbors (KNN); and 6 ensemble-based classifiers—random forest (RF), extra trees (ET), AdaBoost (ADA), GBC, Extreme Gradient Boosting (XGBoost), and Light Gradient Boosting Machine (LGBM).

#### Hyperparameter Handling and Nested Validation

Two hyperparameter strategies were implemented: a fixed configuration using default settings and a tuned configuration using nested CV. In all single-level baselines (stratified 10-fold, group K-fold [K=3], LOPOCV), models used the fixed hyperparameters listed in Table S2 in Multimedia Appendix 1 (right column). Only the nested design performed grid search in the inner loop; no tuning ever used outer-test data. In the fixed configuration, each model was initialized with its default hyperparameters (via a common build_models function), and no grid search was performed. This fixed-parameter setup was used for the 3 single-level CV schemes noted above.

By contrast, the tuned configuration used nested CV, wherein group K-fold (K=3) served as the inner loop for hyperparameter optimization and LOPOCV as the outer loop for evaluation. We chose K=3 to balance 2 constraints. First, each inner training fold required a sufficient number of participants for stable model fitting (approximately 48 of the 71 remaining participants per outer fold). Second, computational cost scales linearly with K in the inner loop and multiplicatively with the 72-fold outer LOPOCV. Hyperparameter tuning was performed using GridSearchCV (scikit-learn) across predefined parameter grids (Table S2 in Multimedia Appendix 1). For each outer fold (held-out participant), the best hyperparameters (denoted θ*) were selected based on inner-loop validation accuracy. A final model was then retrained on the entire outer training set using θ* and evaluated once on the held-out outer participant. This design cleanly decouples tuning from evaluation, minimizing selection bias.

#### Bias-Variance Handling

In the nested configuration, the inner-loop grid spans the parameters that govern model capacity (bias-variance trade-off). Selection maximizes macro-*F*_1_-score averaged across inner folds; when 2 settings are statistically indistinguishable (Δ≤0.5 pp), ties are broken in favor of the simpler configuration (lower capacity) to reduce variance. We quantify variance at evaluation time via outer-fold dispersion across participants and participant-level rank stability.

### CV Methodology

#### Notation and Definitions

To formalize our dataset and modeling pipeline, we define the following: Let the dataset be denoted as *D* = {(*x_i_*, *y_i_*, *p_i_*, *t_i_*)}*^N^_i_*_=1_, where *x_i_* is the feature vector; *y_i_* ∈ {0, 1} is the binary class label; represents the predicted class label for trial *i*; *p_i_* ∈ *P* is the participant identifier; and *t_i_* is the trial number for the *i*th observation within participant *p_i_*.

Let *P* be the set of unique participant IDs with cardinality |*P*|, and *D_p_* = {(*x_i_*, *y_i_*, *t_i_*)} | *p_i_* = *p*} be the set of all trials for participant *p*.

We define *M* as an ML model instance; *θ* ∈ Θ as a specific hyperparameter configuration; Θ as the hyperparameter search space; Train(*M*, *D*_train_) as the training procedure; Eval(*M*, *D*_test_) as the evaluation procedure (eg, accuracy, *F*_1_-score); and Metric(·) as any function computing a classification metric from predicted and true labels.

#### Stratified K-Fold CV

In stratified K-fold CV, the full dataset D is randomly divided into k approximately equal folds while preserving the overall class distribution. We use the stratified variant to maintain class prevalence within each fold; this is not a rebalancing method but a splitting rule that preserves label proportions while remaining trial-wise. For each fold *j* ∈ {1, ..., *k*}, the data are partitioned into a training set *D*^(^*^j^*^)^_train_ and a test set *D*^(^*^j^*^)^_test_. The model is trained on *D*^(^*^j^*^)^_train_, evaluated on *D*^(^*^j^*^)^_test_, and the process is repeated for all folds. Final performance is computed as the average across all folds.

**Figure 2:**
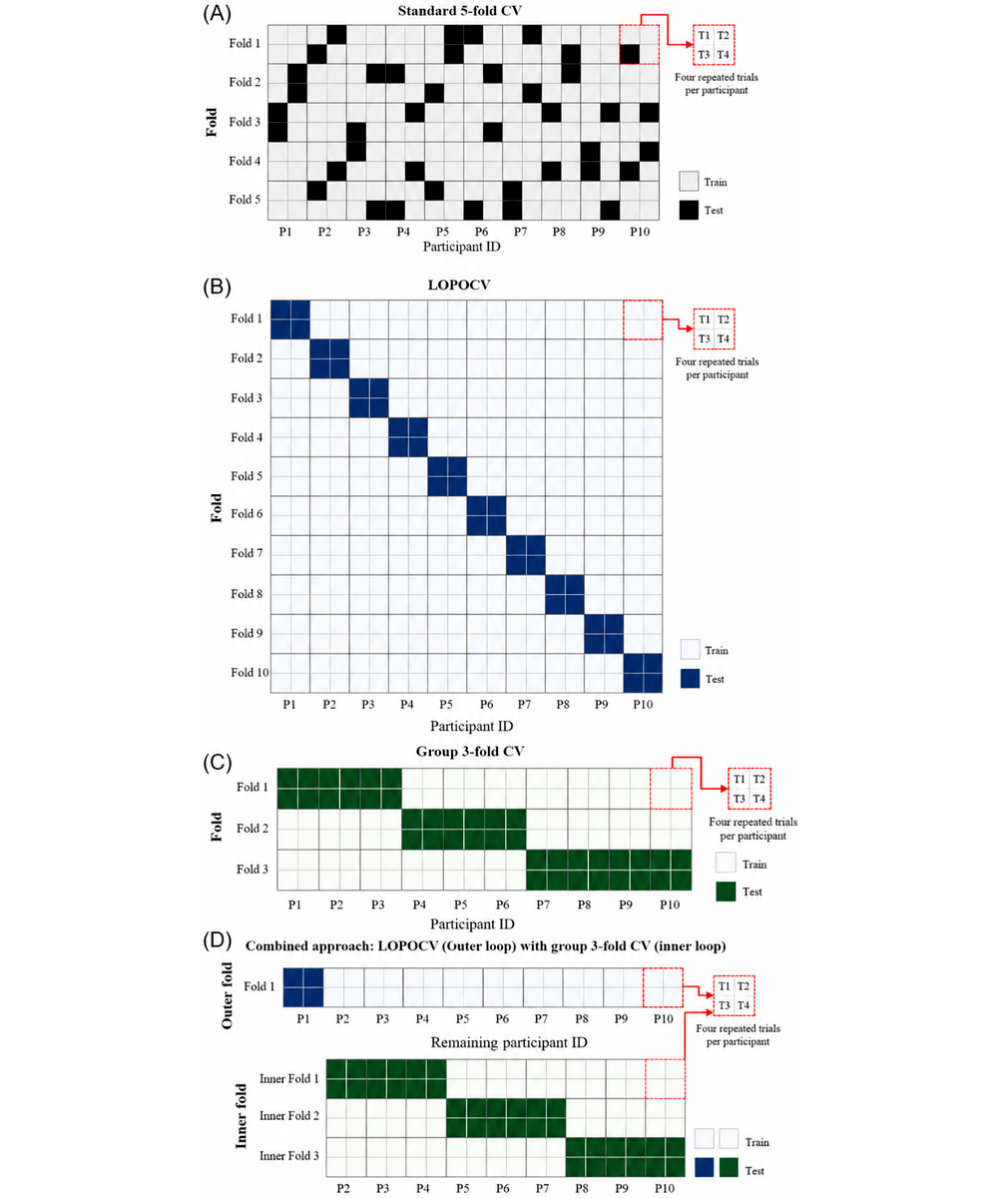
Panels compare 4 evaluation schemes while holding participants as the grouping unit. Tiles represent trials (or segments), colors indicate class labels, and vertical dividers mark participant boundaries. (A) In Trial-wise stratified K-Fold, folds are stratified by label at the trial level, so trials from the same participant may appear in both training and test sets (risk of data leakage). (B) In group K-fold, folds are formed at the participant level, with all trials from a given participant assigned to a single fold, preventing overlap between training and test sets. (C) In leave-one-participant-out (LOPOCV), 1 participant is held out as the test set while the remaining participants form the training set, and this process is repeated across participants. (d) In nested LOPOCV (outer) with group K-fold (inner), the outer loop follows LOPOCV, and within each outer training split, an inner group K-fold is used for hyperparameter tuning; only performance on the outer test sets is reported. Dashed boxes denote outer folds, solid boxes denote inner folds, and arrows illustrate the flow from training to tuning to testing. Vertical grid lines indicate participant boundaries (P1-P10), and the inset shows repeated trials (T1-T4). White indicates training set; green/blue indicates test set for the respective loop. CV: cross-validation.

We used StratifiedKFold (k=10, shuffle=True, random_state=42) from scikit-learn. While commonly used, this method does not control for participant identity and may introduce data leakage in repeated-measures datasets (see Figure 2A). The full pseudocode is provided in Algorithm S1 in Multimedia Appendix 1.

#### Leave-One-Participant-Out Cross-Validation

LOPOCV evaluates true interparticipant generalization by holding out all trials from a single participant in each fold. For each participant *p* ∈ *P*, the model is trained on the dataset *D*^(^*^p^*^)^_train_ = *D*\*D_p_*, where *D_p_* contains all trials from participant *p*. The trained model is then evaluated on *D_p_*, ensuring no overlap between training and testing data.

We implemented LOPOCV using LeaveOneGroupOut from scikit-learn, treating participant ID as the grouping variable. This method enforces participant-level separation but does not address selection bias during hyperparameter tuning (see Figure 2B). An explicit step-by-step description is provided in Algorithm S2 in Multimedia Appendix 1, including participant-averaged metrics and train-test gap calculation.

#### Group K-Fold CV

Group K-fold (K=3) CV preserves participant-level independence by splitting the set of participants *P* into *k* disjoint subsets *P*_1_, ..., *P_k_*. For each fold *j*, all data from participants in the group *P_j_* form the test set *D*^(^*^j^*^)^_test_, while the remaining data constitute the training set *D*^(^*^j^*^)^_train_. The model is trained and evaluated accordingly.

We used GroupKFold (n_splits=3) to ensure that all trials from each participant remained in the same fold. This method maintains participant-level separation during training and testing while allowing multiple participants per fold. Compared with LOPOCV, group K-fold (K=3) offers greater computational efficiency but does not fully isolate tuning from evaluation (see Figure 2C). Implementation details are provided in Algorithm S3 in Multimedia Appendix 1.

#### Combined Approach: Nested LOPOCV (Outer) With Group K-Fold CV (Inner)

To support robust hyperparameter tuning without introducing selection bias, we implemented a nested CV framework. The outer loop used LOPOCV, while the inner loop used group K-fold (K=3) CV for tuning.

In the outer loop, for each participant *p*, the test set *D*^(^*^p^*^)^_outer_test_ comprised all trials from that participant. The remaining data *D*^(^*^p^*^)^_outer_train_=*D*\*D_p_* was used for training and tuning. Within this training set, participants *P*\{*p*} were split into 3 groups for the inner loop.

The inner loop performed a 3-fold CV across these remaining participants. For each inner fold *j*, the training and validation sets were defined as follows:

- Inner validation set:
  *D*_val_^(*p,j*)^ = ∪_*p′∈p(j)*_*D*_*p′*_
- Inner training set:
  *D*_train_^(*p,j*)^ = *D*_outer_train_^(*p*)^\*D*_val_^(*p,j*)^

Each model *M*_θ_^(^*^p^*^,^*^j^*^)^ was trained with a candidate hyperparameter configuration *θ* ∈ Θ and evaluated on *D*_val_^(^*^p,j^*^)^. The optimal configuration θ_p_* was selected based on mean inner validation performance:

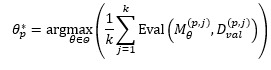



Using θ_p_*, the final model *M*^(^*^p^*^)^ was trained on *D*_outer_train_^(^*^p^*^)^ and evaluated on *D*_outer_test_^(^*^p^*^)^.

This nested design ensures that hyperparameter tuning is fully decoupled from performance estimation by isolating inner tuning folds from outer evaluation folds. Figure 2D illustrates the complete nested validation structure. The nested, participant-aware framework is specified in Algorithm S4 in Multimedia Appendix 1.

The hyperparameter grids used in this process were deliberately designed to be capacity-aware, enabling the inner CV to explicitly explore low-bias/high-variance versus high-bias/low-variance regimes (see Tables S2 and S3 in Multimedia Appendix 1 for the capacity-controlling parameters).

### Performance Evaluation Metrics

We assessed model performance using standard classification metrics derived from the confusion matrix, based on counts of true positives (TPs), true negatives (TNs), false positives (FPs), and false negatives (FNs). The following metrics were computed for each outer test fold in every CV scheme:

Accuracy = (TP + TN)/(TP + TN + FP + FN)

Precision = (TP)/(TP + FP)

Recall = (TP)/(TP + FN)

*F*_1_-score = 2 × (precision × recall)/(precision + recall)

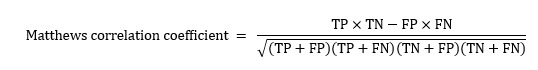

We calculated these metrics from the test-fold predictions of the outer loops for all 4 CV schemes: stratified K-fold, LOPOCV, group K-fold (K=3), and nested LOPOCV.

### Participant Aware Summary Metrics

#### Summary Metrics for Participant-Aware Validation Strategies

For participant-aware strategies (LOPOCV and nested LOPOCV), we computed 2 complementary summaries:

#### Mean LOPOCV Performance (Participant-Averaged)

This metric averages model performance across all participants, giving equal weight to each individual, regardless of the number of trials:

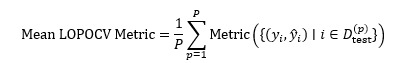



where *P* is the total number of participants; and *D*^(^*^p^*^)^_test_ denotes the outer test set for participant *p*.

#### Overall Performance (Aggregated Level)

This summary pools all test predictions and true labels across outer folds and then computes each performance metric on the combined dataset:

Overall metric = Metric(∪*_p_*_=1_*^P^*{*i* ∈ *D*_test_^(^*^p^*^)^})

The mean LOPOCV performance emphasizes cross-participant stability, whereas overall performance reflects population-level predictive accuracy by weighting participants according to trial count.

### Train-Test Gap: Generalization Stability

To assess generalization stability and potential overfitting, we computed the train-test gap for each outer fold in the LOPOCV and nested CV frameworks. This gap quantifies the difference in model accuracy between the outer training and test sets.

Let θ* ∈ Θ denote the selected hyperparameter configuration—either default (in the fixed configuration) or optimized via inner-loop group K-fold tuning (in nested CV). Let M_θ__*_ be the trained model using this configuration. For each outer fold *f*, corresponding to the held-out participant *p*, the train-test gap is defined as follows:

Gap^(^*^f^*^)^ = Accuracy[M_θ__*_, *D*^(^*^f^*^)^_train_] – Accuracy[M_θ__*_, *D*^(^*^f^*^)^_test_]

This metric quantifies how well a model’s training performance carries over to unseen test participants. Large gaps indicate potential overfitting, whereas small gaps suggest stable generalization.

To summarize overall generalization behavior, we report the mean and SD of the train-test gap across all participants for each model m:

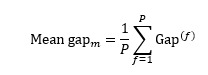



This metric reflects how well training performance translates to unseen test participants. We did not compute the train-test gap for stratified K-fold or group K-fold CV, as both methods allow trials from the same participant to appear in both the training and test sets, thereby violating independence and compromising the interpretability of generalization behavior.

### Bias Estimation Across Validation Strategies

To quantify how CV strategies deviate from participant-aware evaluation, we compared strategy-level summary metrics with a participant-aware baseline. The baseline reference was either LOPOCV or nested LOPOCV, as indicated in each comparison. For each metric *m* ∈ {accuracy, *F*_1_-score, precision, recall, Matthews correlation coefficient}, bias was computed as follows:

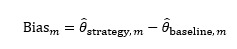



where 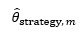 represents the participant-averaged or overall score (as specified) obtained using stratified 10-fold or group K-fold (K=3) CV; and 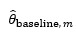 represents the corresponding LOPOCV- or nested LOPOCV–based estimate.

A positive bias implies that the strategy overestimates model performance relative to the participant-aware baseline, whereas a negative bias indicates underestimation. To quantify overall deviation across metrics, we also computed the mean absolute bias (MAB) for each model:

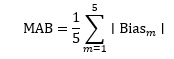



MAB reflects the average magnitude of deviation from the baseline, providing a scalar summary of validation reliability. Larger MAB values indicate greater deviation from a realistic participant-aware estimate and, thus, lower external validity. Lower MAB values indicate that the CV strategy yields results more consistent with robust, participant-separated evaluation.

This procedure is consistent with earlier findings that participant-independent validation frameworks, such as LOPOCV, produce more accurate out-of-sample performance estimates, particularly in domains involving interindividual variability [15,21,22].

### Model Ranking Method

#### Overview

To evaluate the relative performance of classifiers and assess ranking stability across individuals, we implemented a nonparametric model-ranking procedure, following established practices in classifier evaluation [23,24].

Let *A_p,m_* denote the classification accuracy of the model *m* for participant *p*, computed using either LOPOCV or nested LOPOCV. The ranking procedure involved 2 steps.

#### Step 1: Within-Participant Ranking

For each participant *p*, all models were ranked based on their accuracy *A_p,m_*, from highest (rank=1) to lowest (rank=10). Ties were resolved by assigning the average of the spanned ranks.

#### Step 2: Across-Participant Summary Statistics

For each model *m*, we computed the following summary metrics over all participants:

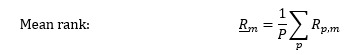



Lower values indicate better average performance across participants.

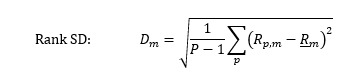



The aforesaid equation reflects consistency of ranking across participants.

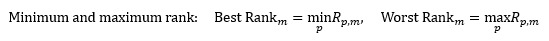



Models with lower mean ranks and smaller rank variability were considered more robust and consistent across individuals. Ranking analyses were conducted independently for LOPOCV and nested CV frameworks to evaluate how hyperparameter tuning influences ranking stability.

### Computational Efficiency Estimation

#### Computational Efficiency Metrics for Model Deployment Feasibility

To assess the feasibility of deploying each model configuration, we evaluated computational efficiency using 3 metrics recorded during outer-loop validation:

#### Training Time Per Outer Fold (Seconds)

Training time per outer fold is defined as the elapsed wall-clock time required to train each model on the outer training set. For nested LOPOCV, this measure includes the full runtime of the inner-loop hyperparameter optimization using GridSearchCV. For LOPOCV, group K-fold (K=3) CV, and 10-fold CV, only the time required for model training (without tuning) was recorded.

#### Inference Time Per Sample (Milliseconds)

Inference time per sample is computed as the average time required to generate a single prediction. This value was averaged over all test samples across outer folds. It indicates the suitability of models for real-time or near–real-time applications.

#### Model Size (Megabytes)

Model size is estimated as the in-memory serialized size of each trained model instance using pickle.dumps(). This metric reflects storage requirements and transferability for deployment in clinical or embedded systems.

All efficiency metrics were automatically logged during model evaluation for each model-CV combination. Comparisons across configurations enable assessment of trade-offs among performance, complexity, and ease of deployment.

### Computational Environment and Reproducibility

All analyses were performed using Python 3.10.12 (Python Foundation) on a high-performance workstation equipped with an Intel Core i9-13900K CPU, an NVIDIA GeForce RTX 4090 GPU, and 64 GB of DDR5 RAM. The software environment included scikit-learn version 1.2.2 [25], XGBoost version 1.4.2 [26], LGBM version 4.3.0 [27], and MLflow 2.10.2 for experiment tracking and reproducibility.

All training runs, CV folds, hyperparameter configurations, and resulting artifacts were managed and logged using MLflow, which provided full experiment lineage and enabled structured comparison across model-validation combinations. Each run recorded parameter settings, metric scores, training durations, and serialized model outputs, enabling seamless integration with the reported analysis.

Randomized operations, including data shuffling, CV splitting, and model initialization, were controlled using a fixed seed (random_state=42). All splitters and estimators were instantiated with this seed where applicable; CV groups were specified as participant IDs to enforce participant integrity.

## Results

### Model Performance Across Validation Strategies

Table 2 presents overall and participant-averaged accuracy for each classifier across 4 validation strategies. Performance varied by model type and validation scheme.

**Table 2:** Model performance across validation strategies.

Model	LOPOCV^a^ (overall)	LOPOCV, mean (SD)^b^	Nested CV^c^ (overall)	Nested CV, mean (SD)^b^	Group CV (SD)	10-fold CV
k-Nearest neighbors	0.69	0.69 (0.29)	0.68	0.68 (0.30)	0.65 (0.03)	0.91
Logistic regression	0.64	0.64 (0.35)	0.64	0.64 (0.35)	0.63 (0.03)	0.7
AdaBoost	0.64	0.63 (0.34)	0.63	0.61 (0.34)	0.63 (0.03)	0.77
Linear discriminant analysis	0.63	0.63 (0.35)	0.63	0.63 (0.35)	0.63 (0.03)	0.7
Quadratic discriminant analysis	0.66	0.66 (0.34)	0.66	0.66 (0.35)	0.67 (0.03)	0.77
Gradient Boosting Classifier	0.68	0.67 (0.31)	0.66	0.66 (0.32)	0.65 (0.03)	0.85
Extra trees	0.66	0.67 (0.34)	0.65	0.66 (0.33)	0.64 (0.04)	0.91
Random forest	0.65	0.64 (0.34)	0.65	0.65 (0.34)	0.64 (0.04)	0.85
Extreme Gradient Boosting	0.65	0.64 (0.33)	0.67	0.66 (0.33)	0.65 (0.03)	0.87
Light Gradient Boosting Machine	0.63	0.62 (0.33)	0.64	0.62 (0.33)	0.62 (0.03)	0.79

^a^LOPOCV: leave-one-out cross-validation.

^b^LOPOCV, mean (SD), and nested CV, mean (SD), refer to participant-averaged accuracy.

^c^CV: cross-validation.

The highest accuracy values for the ensemble models ET, GBC, RF, and XGBoost were obtained under 10-fold CV. For example, ET achieved 0.91 accuracy under 10-fold CV and 0.66 under LOPOCV. LR and LDA exhibited relatively stable accuracy across validation methods, with differences ≤0.03 between LOPOCV and 10-fold CV.

Accuracy estimates under nested CV closely matched those from LOPOCV, differing by ≤0.02 across all classifiers. Group K-fold (K=3) CV produced intermediate accuracy scores, typically falling between LOPOCV and 10-fold CV. Full evaluation metrics (accuracy, *F*_1_-score, precision, recall, and Matthews correlation coefficient) for each model are provided in Table S5 in Multimedia Appendix 1.

### Train-Test Gap and Overfitting Risk

**Figure 3:**
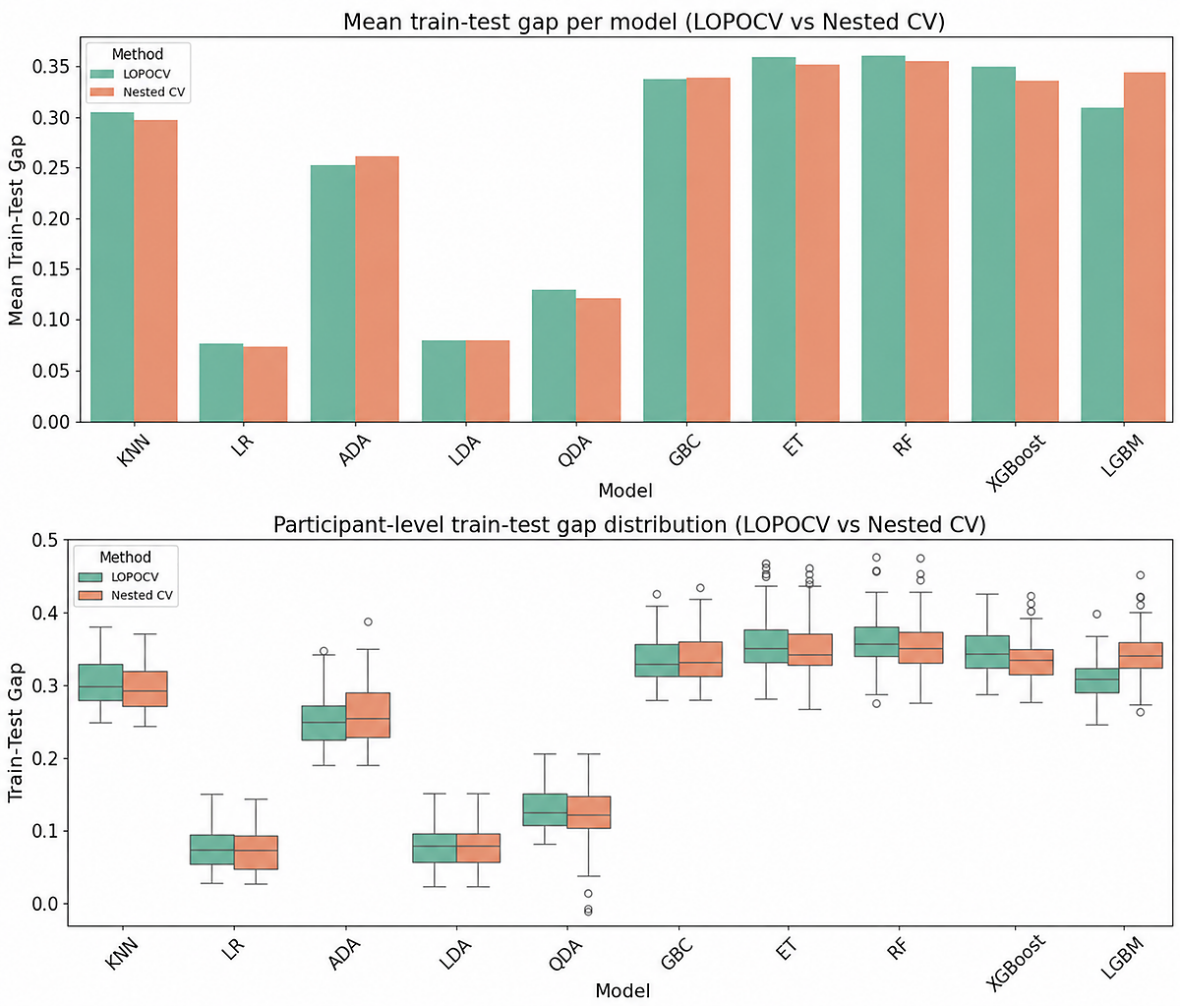
Generalization gap under participant-aware validation. (A) Mean train-test gap for each classifier under leave-one-out cross-validation (LOPOCV) and nested LOPOCV; bars represent the mean across participants and error bars indicate SD, with smaller gaps reflecting better generalization. (B) Participant-level dispersion of the train-test gap for the same classifiers and validation schemes, shown as boxplots (center line indicates median; box represents IQR; whiskers are 1.5×IQR; points indicate outliers). Wider IQRs and more frequent outliers—particularly for ensemble models—indicate greater instability in participant-level generalization. Complete numerical results for all models and both schemes are provided in Table S4 in Multimedia Appendix 1. ADA: AdaBoost; AUC: area under the curve; CV: cross-validation; ET: extra trees; GBC: Gradient Boosting Classifier; KNN: k-nearest neighbors; LDA: linear discriminant analysis; LGBM: Light Gradient Boosting Machine; LR: logistic regression; QDA: quadratic discriminant analysis; RF: random forest; XGBoost: Extreme Gradient Boosting.

Figure 3A summarizes the mean (SD) train-test gaps for each classifier under LOPOCV and nested LOPOCV; exact values are provided in Table S4 in Multimedia Appendix 1. Gaps ranged from 0.08 to 0.36 across models. Ensemble methods (ET, RF, and GBC) showed the largest gaps (≥0.34) under both validation strategies, whereas LR and LDA were approximately 0.08. Gap estimates were similar for LOPOCV and the nested scheme, with no consistent differences across models. Figure 3B shows participant-level distributions. Ensemble models displayed wider IQRs and more outliers, whereas linear models were more tightly clustered around their medians, consistent with Figure 3A.

**Figure 4:**
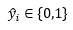
Inline graphic 1.

### Bias Between Validation Strategies

Bias (Δ) for all classifiers is reported in Table S6 in Multimedia Appendix 1 and visualized in Figure S1 in Multimedia Appendix 1 (diverging scale centered at 0). Across classifiers, 10-fold CV exhibits the largest positive Δ relative to participant-aware baselines, consistent with performance inflation when trials from the same participant appear in both training and test sets. By contrast, LOPOCV, nested LOPOCV, and group K-fold yield Δ values approximately 0 across metrics. For example, KNN shows marked increases relative to LOPOCV under 10-fold CV (accuracy +0.22, Matthews correlation coefficient +0.82, MAB 0.36 in Table S6 in Multimedia Appendix 1), whereas LR shows no measurable difference between nested LOPOCV and Group K-fold.

### Model Ranking Consistency Across Participants

Table 3 summarizes participant-level rank statistics for each classifier under LOPOCV and nested CV. For each model, the table displays the mean rank, SD, best rank, and worst rank across all participants. Lower rank values correspond to higher participant-level accuracy. Across both validation strategies, ET yielded the lowest mean ranks (5.19 for LOPOCV and 5.39 for nested CV), whereas LGBM had the highest (6.05 and 6.06, respectively). SDs ranged from 1.89 (RF, LOPOCV) to 2.79 (ADA, nested CV).

**Table 3:** Model ranking statistics under leave-one-participant-out cross-validation rank and nested cross-validation^a^.

Model	Leave-one-participant-out cross-validation rank				Nested cross-validation rank		
	Mean (SD)^b^	Best	Worst	Mean (SD)^b^		Best	Worst
k-Nearest neighbors	5.39 (2.65)	1	10	5.34 (2.63)		1	10
Logistic regression	5.51 (2.70)	1	10	5.42 (2.70)		1	10
AdaBoost	5.64 (2.57)	1	10	5.81 (2.79)		1	10
Linear discriminant analysis	5.55 (2.70)	1	10	5.52 (2.74)		1	10
Quadratic discriminant analysis	5.28 (2.43)	1	10	5.27 (2.52)		1	10
Gradient Boosting Classifier	5.22 (2.15)	1	9	5.47 (2.24)		1	10
Extra trees	5.19 (2.16)	1	10	5.39 (2.02)		2	10
Random forest	5.49 (1.89)	1.5	10	5.37 (2.06)		1	10
Extreme Gradient Boosting	5.68 (2.48)	1	10	5.37 (2.23)		1	10
Light Gradient Boosting Machine	6.05 (2.34)	1.5	10	6.06 (2.14)		2	10

^a^Lower mean ranks and SDs indicate more consistent participant-level performance. Rankings were based on individual classification accuracy within each outer test fold.

^b^Best-rank values were fractional (eg, [1+2]/2=1.5) when multiple models achieved identical participant-level accuracy and tied for top performance. In such cases, ranks were averaged according to standard ranking conventions [23].

All classifiers achieved a best rank of 1.0 for at least one participant, with fractional values (eg, 1.5) indicating ties averaged according to standard conventions. The worst rank was uniformly 10.0 for all classifiers. Differences in rank between LOPOCV and nested CV were small. For 8 of the 10 models, the absolute difference in mean rank was ≤0.25. Changes in rank variability (SD) were also limited, with maximum observed shifts of 0.31 in mean rank (XGBoost) and 0.22 in SD (ADA).

### Computational Efficiency Analysis

Table 4 presents the average training time per outer fold, inference time per sample, and final model size for each classifier under 4 validation strategies. Training time varied by more than 4 orders of magnitude across models. The fastest models—KNN, QDA, LDA, and LR—completed training in under 0.02 seconds per fold across all validation strategies. LGBM required 275 seconds per fold under nested CV and 125 seconds under 10-fold CV, even without tuning. XGBoost was more efficient than the other 3 ensembles during tuning, requiring approximately 3.7 seconds per fold.

**Table 4:** Training time, inference latency, and model size by classifier and cross-validation strategy.

Model	Training time (seconds)^a^					Inference time (ms/sample)^b^					Model size (MB)^c^			
	10-fold CV^d^	Group CV	LOPOCV^e^	Nested CV^f^	10-fold CV		Group CV	LOPOCV	Nested CV^f^	10-fold CV		Group CV	LOPOCV	Nested CV^f^
k-Nearest neighbors	0.012	0.012	0.012	0.026	0.00^g^		0.00	0.00	0.00	0.101		0.074	0.110	0.110
Logistic regression	0.013	0.013	0.013	0.018	0.00		0.00	0.00	0.00	0.001		0.001	0.001	0.001
AdaBoost	0.084	0.077	0.094	0.183	0.00		0.00	0.00	0.00	0.026		0.026	0.026	0.032
Linear discriminant analysis	0.013	0.012	0.013	0.016	0.00		0.00	0.00	0.00	0.002		0.002	0.002	0.002
Quadratic discriminant analysis	0.012	0.012	0.012	0.016	0.00		0.00	0.00	0.00	0.003		0.003	0.003	0.003
Gradient Boosting Classifier	0.157	0.133	0.165	0.604	0.00		0.00	0.00	0.00	0.110		0.108	0.110	0.231
Extra trees	0.138	0.141	0.141	0.476	0.00		0.00	2.00	2.00	3.053		2.125	3.285	3.980
Random forest	0.327	0.308	0.326	0.600	0.00		0.00	2.00	2.00	1.728		1.240	1.849	1.449
Extreme Gradient Boosting	1.268	1.317	1.228	3.708	0.00		0.00	0.00	0.00	0.211		0.208	0.211	0.201
Light Gradient Boosting Machine	125.24	95.41	143.22	275.48	0.00		0.00	0.00	0.00	0.214		0.163	0.232	0.201

^a^Training time reflects the mean wall-clock duration per fold.

^b^Inference time is reported per sample and rounded to the nearest millisecond.

^c^Model size is based on the serialized object in memory.

^d^CV: cross-validation.

^e^LOPOCV: leave-one-out cross-validation.

^f^Nested CV includes tuning overhead via GridSearchCV.

^g^<0.005 ms/sample.

Inference times were uniformly low. All models classified a test sample in ≤2 milliseconds per sample. ET and RF had nonzero inference times (2 ms), but all models were well within real-time application thresholds. Model sizes remained modest. LR had the smallest footprint (~1 kB). ET under nested CV had the largest size (~4 MB), followed by RF and GBC. LGBM maintained a small model size (~0.23 MB) despite its long training time.

## Discussion

### Principal Findings

The main finding of this study is that CV strategy substantially influences reported model performance in repeated-measures classification. Stratified 10-fold CV systematically overestimated accuracy (eg, ET: 0.91 vs 0.66 under LOPOCV) due to participant-level data leakage. Participant-aware strategies (LOPOCV, group K-fold, and nested CV) produced more conservative and stable estimates. The nested LOPOCV + group K-fold framework achieved the best balance among unbiased evaluation, participant-aware separation, and hyperparameter tuning decoupling, with minimal additional bias compared with standalone LOPOCV.

### Theoretical Rationale for Participant-Aware Validation

Accurate model evaluation in repeated-measures datasets requires validation strategies that respect the nested data structure, specifically the dependency among trials within each participant. Classical CV methods assume independent and identically distributed observations, an assumption that is violated when multiple measurements come from the same individual.

Stratified K-fold CV maintains class balance across folds but splits individual trials randomly, often placing multiple trials from the same participant in both the training and test sets. This introduces participant-specific information leakage, which in turn inflates reported performance metrics and misrepresents generalizability [8,10,15].

LOPOCV addresses this leakage by holding out all trials from a single participant in each fold. This ensures participant-level independence during evaluation [8]. However, LOPOCV does not prevent selection bias: if hyperparameter tuning uses data from the same participants later used for evaluation, model selection remains compromised. Group K-fold (K=3) CV offers a partial solution by assigning all trials from a participant to the same fold; however, when used for both tuning and evaluation, it still allows indirect bias to persist.

To resolve both leakage and selection bias, this study implemented a nested CV structure: LOPOCV for outer-loop evaluation and group K-fold (K=3) for inner-loop tuning. This structure ensures that evaluation is based on participants unseen during training and tuning. Furthermore, tuning leverages repeated trials from different individuals, handling intrasubject variability without reintroducing test data. This approach aligns with established recommendations for unbiased evaluation in supervised learning [15,16,28].

A key rationale behind this design lies in recognizing that, in repeated-measures contexts, the effective sample size approximates the number of participants, not the total number of trials [29,30]. Trial-level CV methods that ignore this dependency systematically underestimate generalization error and lead to inflated performance metrics, particularly for models that exploit individual-specific patterns.

### Effects of Validation Strategy on Model Performance

Stratified 10-fold CV produced the highest reported accuracies but inflated generalization estimates, with differences of up to 0.25 compared with LOPOCV (eg, ET: 0.91 vs 0.66). Among participant-aware methods, high-capacity classifiers (KNN, ET, and GBC) still exhibited train-test gaps ≥0.30 under standalone LOPOCV, suggesting overfitting to participant-idiosyncratic patterns when tuning was not decoupled from evaluation.

Group K-fold (K=3) preserved participant identity while reducing computational burden, with performance comparable to LOPOCV and stable rankings. Nested CV produced the most cautious and consistent estimates across classifiers: it minimized validation bias, narrowed train-test gaps, and maintained ranking consistency, yielding a representative estimate of out-of-sample performance. A related SRSH study using participant-wise evaluation reported 75.6% accuracy, which aligns with the participant-aware range observed here and contrasts with the optimistic trial-wise figures [14].

These findings answer research questions 1 and 2:

- Research question 1: LOPOCV reduces leakage but may not prevent overfitting when intrasubject variability is not managed.
- Research question 2: A nested participant-aware framework provides the most robust and unbiased evaluation of model generalization in repeated-measures data.

### Model Stability and Ranking Consistency

Although the top classifiers showed comparable average accuracies, their rankings differed considerably at the individual participant level. Specifically, ET and RF demonstrated greater ranking stability across participants compared with LGBM and ADA, which showed higher variability. Despite these individual differences, rankings remained highly stable between LOPOCV and nested CV. For 8 out of 10 models, the absolute difference in mean rank between the 2 schemes was ≤0.25, and the change in rank SD was ≤0.17, supporting the conclusion that participant-aware tuning does not distort model comparisons.

These findings directly address research question 3, emphasizing the practical value of incorporating participant-level validation frameworks when deploying ML models in real-world scenarios. While model stability is essential, computational demands must also be considered when selecting validation strategies for deployment.

### Efficiency and Practical Considerations

Validation strategies varied considerably in computational cost. Nested CV incurred the highest training time, especially for ensemble models (eg, LGBM: >275 seconds/fold), due to inner-loop hyperparameter tuning. By contrast, linear models (eg, LR and LDA) trained in under 0.02 seconds per fold, underscoring the substantial overhead introduced by nested designs. Group K-fold (K=3) CV offered a computationally efficient compromise, producing performance estimates comparable to LOPOCV with minimal bias while avoiding the full tuning cost of nested CV.

Despite differences in training time, all models exhibited low inference latency (≤2 ms/sample) and small memory footprints, making them suitable for deployment in real-time settings once trained. In practice, training efficiency—not inference cost—should guide validation strategy selection, particularly in clinical or iterative modeling contexts where scalability and runtime constraints are critical.

### Limitations and Future Work

This study was designed to isolate the impact of validation strategy on performance estimation rather than to maximize predictive accuracy. Several limitations should be acknowledged. First, the analysis was restricted to manually engineered features and narrow hyperparameter grids. While this ensured consistency across models, broader, systematically documented optimization techniques, such as Bayesian search or automated feature extraction, may yield different performance rankings. Second, the dataset was limited to a binary classification task in 72 individuals (43 males and 29 females; mean age 28.4 years, SD 6.7 years) following ACLR with hamstring autograft, all recruited 6-24 months after surgery. The extent to which the observed validation discrepancies generalize to cohorts with different demographic profiles (eg, slightly older populations, different graft types, time since surgery, or varying preinjury activity levels) remains an open question. Future studies should also test generalizability in multiclass problems, longitudinal predictions, and additional clinical contexts. Third, although the study included a range of linear and ensemble learning algorithms, it did not evaluate deep neural networks, which may be more sensitive to overfitting and leakage in repeated-measures data. Applying participant-aware nested validation to recurrent or convolutional neural networks, particularly in high-dimensional time-series biomechanics, remains a critical direction for future research. Fourth, this study did not address probability calibration or algorithmic fairness, both of which are essential for the deployment of clinical ML. Future work should integrate calibration metrics (eg, Brier score, calibration curves) and evaluate fairness-aware performance under participant-aware validation schemes. Finally, feature-level interpretation was out of scope. We focused on evaluating models rather than identifying which features drive predictions. Importance estimates depend on the validation design and may be unstable under trial-wise splits. We therefore reserve feature attribution for future work conducted strictly under the nested, participant-aware protocol established here, ensuring that any reported importances reflect leakage-free evaluation.

### Comparison With Previous Literature

The limitations of stratified CV in repeated-measures settings have been well documented. Early theoretical work identified selection bias and performance inflation when training and evaluation are not properly separated [9,15]. These studies laid the foundation for participant-aware evaluation, though they did not explicitly address repeated measures or participant-specific dependency structures.

More recent applied studies in neuroimaging, biomechanics, and mobile health have adopted LOPOCV or group CV to control for participant-level leakage (eg, [8,9]). However, many of these implementations lack full tuning-evaluation decoupling, often using the same CV scheme for both hyperparameter selection and final testing. This results in partial leakage and residual bias, even when participants are held out during the final evaluation.

Prior work has noted the risk of data leakage from trial-wise CV and the value of participant-aware evaluation [8,9,15]. However, no study has systematically benchmarked a fully nested CV design (LOPOCV outer loop or group K-fold inner loop) for repeated-measures classification in biomechanics across multiple classifiers and evaluation dimensions. This study provides such a benchmark, quantifying classification performance, validation bias, train-test generalization, participant-level ranking stability, and computational efficiency across 4 strategies and 10 classifiers. A comparative overview of previous methodologies and their limitations is provided in Table 5, highlighting how this study addresses critical gaps in the current literature.

**Table 5:** Summary of prior studies on CV^a^ strategies in repeated-measures machine learning.

Study	Domain	Validation methods	Key contribution	Limitations
Varma and Simon [15]	Bioinformatics	Nested vs nonnested CV	Theoretical analysis of overfitting and bias	Did not address repeated measures or participant-dependent data
Cawley and Talbot [16]	Machine learning theory	Model selection bias	Highlighted the need for a nested CV in selection	No empirical comparison of CV types
Krstajic et al [31]	Chemoinformatics	K-fold and CV pitfalls	Explained how improper CV inflates model complexity	No participant separation or tuning evaluation
Sohrab et al [9]	Digital mental health	K-fold vs LOPOCV^b^	Demonstrated leakage risk in repeated trials	Did not evaluate tuning or rank stability
Steyerberg and Harrell [32]	Model development	Bootstrapping	Advocated internal/external validation	Not focused on repeated-measures structure
Varoquaux et al [8]	Neuroimaging	LOPOCV and nested CV	Advocated for subject-aware design in brain decoding	Limited metric scope; no bias, time, or rank evaluation
Neto et al [33]	Digital health diagnostics	Permutation tests and CV	Detected identity confounding due to improper CV	Proposed correction, but no comparative benchmarking
Xu and Goodacre [34]	General machine learning	CV, bootstrapping, and systematic	Compared methods for small-sample generalization	Did not address participant-dependent data
Heo et al [35]	Stroke outcomes	Retrospective CV	Compared multiple machine learning models for prediction	No subject-aware splits or nested tuning
Dehghani et al [36]	Human activity recognition	Record-wise vs subject-wise CV	Subject-wise CV prevents leakage and improves realism	Did not include ranking or tuning comparisons
Ferdinandy et al [37]	Animal behavior	K-fold and leave-one-out cross-validation	Showed that correlation in behavior data skews CV estimates	No nested tuning; focused on exploratory data
Islam et al [38]	Genomic prediction	5-fold across cycles	Incorporated repeated cycles in prediction	Did not assess CV design effects systematically
Wilimitis and Walsh [22]	Clinical machine learning	Group CV and LOPOCV	Tutorial on subject-aware evaluation	No computational benchmarking or model ranking
Lu et al [39]	Postsurgical infection	Bootstrapping + K-fold	Empirical uncertainty analysis of prediction	No subject-level CV or overfitting assessment
Chen et al [40]	Surgical infection	Calibration + CV	Evaluated clinical machine learning predictions with calibration	Lacked focus on repeated measures or leakage
Ghasemzadeh et al [41]	Speech and hearing	K-fold and nested CV	Nested CV improved generalization and sample efficiency	No participant-level data structure handling
Lumumba et al [42]	General machine learning	Leave-one-out cross-validation, K-fold, and repeated K-fold	Repeated K-fold improved stability in imbalanced data	Did not address subject-specific leakage
This study	Biomechanics/clinical machine learning	10-fold, group CV, LOPOCV, and nested LOPOCV	Jointly evaluates subject-aware nested CV with bias, generalization, model ranking, and efficiency metrics	Benchmarking subject-aware validation for repeated-measures movement data under real-world constraints

^a^CV: cross-validation.

^b^LOPOCV: leave-one-out cross-validation.

### Regulatory Relevance

Subject-aware validation aligns with emerging regulatory expectations for clinical ML systems, although direct regulatory conclusions should not be drawn from a single case study. The nested CV framework demonstrated here ensures subject-aware separation in both evaluation and hyperparameter tuning, reducing the risk of performance inflation, an issue highlighted in regulatory and ethical reviews of artificial intelligence (AI) systems. Several regulatory documents and frameworks explicitly support these principles:

- TRIPOD+AI [17] calls for complete and reproducible reporting of validation procedures that mimic intended use scenarios.
- The FDA’s Good Machine Learning Practice guidelines [43] stress the need for strict separation between training and test data, comprehensive documentation of data lineage, and bias minimization.
- The EU Medical Device Regulation (2017/745) [44] and the proposed Artificial Intelligence Act (2023) [45] categorize clinical AI tools as high-risk technologies, requiring developers to present robust performance evidence and demonstrate algorithmic transparency.

By aligning with these standards, the nested CV approach not only enhances internal validity but also contributes to regulatory readiness. It provides a principled validation pathway aligned with international expectations for safety, accountability, and trustworthiness in AI-driven medical tools.

### Summary of Implications

This study demonstrates that the validation strategy critically influences model trustworthiness in repeated-measures classification. Trial-level CV overstates performance by ignoring participant dependencies, while subject-aware methods, particularly nested CV, offer more realistic and unbiased estimates of generalization. When full nesting is computationally infeasible, group K-fold (K=3) CV offers a practical alternative, maintaining participant-level separation with minimal validation bias. In deployment scenarios, where model outputs influence clinical or behavioral decisions, variability in participant-level predictions may undermine reliability.

While this study used balanced class distributions, real-world applications often involve class imbalance and prevalence shifts. In such contexts, metrics such as positive predictive value and negative predictive value may vary despite stable sensitivity and specificity [46]. Future implementations should consider calibration and context-specific performance metrics to ensure reliability under deployment conditions.

### Conclusions

This study highlights the critical role of validation strategy in evaluating ML models trained on repeated-measures data. Through a comparative evaluation of 4 CV methods—stratified 10-fold CV, group K-fold (K=3) CV, LOPOCV, and nested LOPOCV—we demonstrate that standard approaches often overestimate model performance by failing to account for participant-level dependencies.

Nested, subject-aware validation provided the most reliable generalization estimates in this repeated-measures setting, and our efficiency analysis indicates viable lower-cost options when full nesting is impractical. Classifier stability, train-test gap, and computational efficiency were all found to vary substantially across validation schemes, showing that validation design materially affects performance estimates relevant for deployment.

These results have practical implications for clinical model development, where decisions based on inflated metrics may compromise safety and reproducibility. The nested subject-aware framework proposed here offers a principled path toward transparent and trustworthy ML systems aligned with current regulatory expectations in health care and other domains characterized by repeated-measures designs. Subject-aware validation is not merely a methodological refinement but a practical necessity for developing reproducible, generalizable, and deployment-ready ML systems in biomechanics, behavioral research, and clinical monitoring.

## Data Availability

The datasets analyzed during this study are not publicly available due to Swedish legislation regarding patient safety and medical confidentiality; however, deidentified data are available from the corresponding author (FA) on reasonable request, subject to jurisdiction. The complete codebase and MLflow logs are publicly available online [47].

## Appendix

Multimedia Appendix 1 TRIPOD+AI (Transparent Reporting of a multivariable prediction model for Individual Prognosis Or Diagnosis—Artificial Intelligence) compliance checklist and additional analysis.

## Abbreviations

ACLR: anterior cruciate ligament reconstruction
ADA: AdaBoost
AI: artificial intelligence
CV: cross-validation
ET: extra trees
FN: false negative
FP: false positive
GBC: Gradient Boosting Classifier
KNN: k-nearest neighbors
LDA: linear discriminant analysis
LGBM: Light Gradient Boosting Machine
LOPOCV: leave-one-out cross-validation
LR: logistic regression
MAB: mean absolute bias
ML: machine learning
QDA: quadratic discriminant analysis
RF: random forest
SRSH: standardized rebound side hops
TN: true negative
TP: true positive
TRIPOD+AI: Transparent Reporting of a multivariable prediction model for Individual Prognosis Or Diagnosis—Artificial Intelligence
XGBoost: Extreme Gradient Boosting
