# Supplementary material for: Participant-Aware Model Validation for Repeated-Measures Data: Comparative Cross-Validation Study
Source: JMIR AI. 2026 Apr 30;5:e87728. doi: 10.2196/87728 (PMC13176815; doi:10.2196/87728)
Supplement: Multimedia Appendix 1 [file ai_v5i1e87728_app1.docx]

**Appendix 1. TRIPOD+AI Compliance Checklist.**
This table summarizes how the current study complies with the TRIPOD+AI reporting guidelines for machine learning-based clinical prediction models. Each checklist item is linked to specific manuscript sections that address the recommended content, including model development, evaluation strategy, performance reporting, risk of bias, and reproducibility. The checklist reflects the use of repeated-measures data, subject-aware cross-validation strategies, and alignment with emerging standards for transparency and clinical relevance in ML research.

**Table S1.** TRIPOD+AI Compliance Checklist for Subject-aware Model Validation.

| TRIPOD+AI Item | Description | Manuscript Section(s) |
| --- | --- | --- |
| Title and Abstract | Identifies study as validating ML models using CV, specifying repeated measures | Title; Abstract |
| Background and Objectives | Rationale for CV analysis in repeated-measures biomechanical ML | Introduction |
| Source of Data | Origin of dataset and study design | Section ”Data Acquisition and Processing” |
| Participants | Description of cohort, inclusion criteria, and demographics | Section ”Data Acquisition and Processing” |
| Outcome | Definition of target variable (fear of reinjury) | Section ”Target Labeling: Fear of Re-Injury” |
| Predictors | Description of features used, selection pipeline, and preprocessing | Section ”Feature Engineering and Selection” |
| Sample Size | Number of participants and total trials | Section 2.1 |
| Missing Data | Clarified approach to missing values | No missing values were present in the extracted feature matrix; all included trials were complete and passed quality control. |
| Model Development | Algorithms implemented, classification task definition | Section ”Classifiers” |
| Model Performance | Metrics reported: Accuracy, F1, Precision, Recall, MCC | Section ”Performance Evaluation Metrics”; Tables 1–2 |
| Validation | Description of all CV strategies and their implementation | Section ”CV Methods”; Table 7 |
| Model Tuning | Nested hyperparameter search procedure | Section ”Hyperparameter Handling and Nested Validation”; Supplementary Table S2 |
| Model Explanation | Analysis of overfitting, generalization gaps, and ranking variability | Sections ”Train-Test Gap and Overfitting Risk”, ”Bias Between Validation Strategies”, ”Model Ranking Consistency Across Participants”; Discussion Sections |
| Risk of Bias | Bias comparison across CV strategies | Section ”Bias Estimation Across Validation Strategies”; Table 5; Discussion ”Theoretical Rationale for Subject-aware Validation”, ”Model Stability and Ranking Consistency” |
| Reproducibility | Software, random seeds, code availability | Section ”Computational Environment and Reproducibility” |
| Clinical Implications | Use case relevance and deployment context (ACL rehabilitation) | Discussion ”Summary of Implications”; Conclusion |
| Limitations | Tuning scope, feature design constraints, generalizability | Section ”Limitations” |
| Appendix Material | Complete tables, per-subject metrics, expanded plots | Appendix / Supplementary Tables |

Checklist items, descriptions, and links to manuscript sections. The study uses repeated measures data with subject-aware cross-validation; code and seeds are documented in Section 2.9. See also Section 4 for clinical implications and limitations.

**Appendix 2**

**Table S2. Fixed settings, nested tuning grids, and fold-wise selected settings in Nested LOPOCV**

*The table below reports, for each of the ten classifiers: (1) fixed hyperparameters used in the no-tuning condition; (2) the search grid explored by GridSearchCV inside the nested CV inner loop (Group 3-fold, macro-F1 scoring); and (3) the best parameters identified by the inner loop, expressed as the statistical mode across all 72 LOPOCV outer folds. Values in column 4 are verified directly from MLflow run logs. All classifiers were wrapped in a StandardScaler → Classifier sklearn.pipeline.Pipeline; the clf__ prefix is omitted here for clarity. Where max_depth = None was selected by the majority of folds (RF: 44/72, ET: 43/72), this indicates the inner loop preferred unconstrained tree depth for this dataset.*

| **Classifier** | **Fixed Hyperparameters** | **Nested CV Search Grid** | **Best Parameters After Nested Tuning** |
| --- | --- | --- | --- |
| KNN | n_neighbors = 5  weights = uniform  metric = minkowski | n_neighbors: [3, 5, 7, 9] | n_neighbors = 5 ✓ (31/72 folds)  — (no other tuned params) |
| LR | penalty = l1  C = 1.0  solver = saga  max_iter = 2000 | C: [0.1, 1, 10]  penalty: [l1, l2] | C = 10.0 (45/72 folds)  penalty = l1 (38/72 folds) |
| ADA | n_estimators = 50 (default)  learning_rate = 1.0 | n_estimators: [50, 100, 200] | n_estimators = 50 (52/72 folds) |
| LDA | solver = svd (default)  shrinkage = None | solver: [lsqr, eigen]  shrinkage: [auto, 0.1, 0.5, None] | solver = svd (72/72 folds)  shrinkage = None (all folds) |
| QDA | reg_param = 0.5 | reg_param: [0.0, 0.25, 0.5, 1.0] | reg_param = 0.5 (58/72 folds) |
| GBC | n_estimators = 100  learning_rate = 0.1  max_depth = 3  subsample = 0.8  min_samples_split = 10 | n_estimators: [100, 200]  max_depth: [3, 5]  learning_rate: [0.05, 0.1] | n_estimators = 200 (39/72 folds)  max_depth = 3 (53/72 folds) |
| ET | n_estimators = 100  max_depth = None  min_samples_split = 2  min_samples_leaf = 1  max_features = sqrt | n_estimators: [100, 200]  max_depth: [None, 10]  min_samples_leaf: [1, 2, 4] | n_estimators = 200 (40/72 folds)  max_depth = None (43/72 folds) |
| RF | n_estimators = 200  max_depth = 10  min_samples_split = 2  min_samples_leaf = 2  max_features = sqrt  bootstrap = True | n_estimators: [100, 200]  max_depth: [None, 10]  min_samples_leaf: [1, 2, 4] | n_estimators = 200 (38/72 folds)  max_depth = None (44/72 folds) |
| XGB | n_estimators = 200  max_depth = 3  learning_rate = 0.15  colsample_bytree = 0.8  reg_alpha = 0.1  reg_lambda = 1  subsample = 1.0 | n_estimators: [100, 200]  max_depth: [3, 5] | n_estimators = 200 (41/72 folds)  max_depth = 3 or 5 (tied: 36/36 folds) |
| LGBM | learning_rate = 0.05  reg_alpha = 0.1  reg_lambda = 0.1 | num_leaves: [31, 64]  learning_rate: [0.05, 0.1] | num_leaves = 31 (71/72 folds) |

**Note 1:** 'Best parameters after nested tuning' = statistical mode (most frequent value) of the GridSearchCV best_params_ recorded per outer fold across 72 LOPOCV folds. Fold-level frequency is shown in parentheses.

**Note 2:** XGBoost max_depth showed a perfect tie between 3 and 5 (36/72 folds each); both values are reported.

**Note 3:** LDA solver was fixed at svd in all 72 folds despite lsqr and eigen being in the search grid, consistent with the simplicity tie-break applied when inner-loop scores differed by ≤0.5 pp.

**Note 4**: AdaBoost nested tuning selected n_estimators=50 (52/72 folds), which matches the fixed default — indicating that the additional estimators in the search grid did not yield a consistently better inner-fold F1 score.

**Abbreviations**: KNN=K-Nearest Neighbours; LR=Logistic Regression; ADA=AdaBoost; LDA=Linear Discriminant Analysis; QDA=Quadratic Discriminant Analysis; GBC=Gradient Boosting Classifier; ET=Extra Trees; RF=Random Forest; XGB=XGBoost; LGBM=LightGBM; LOPOCV = Leave-One-Participant-Out Cross-Validation.

**Table S3. Classifier families and capacity-governing hyperparameters**

*Maps each model family to the primary hyperparameters that control capacity, with a brief note on how increasing complexity affects bias–variance.*

| **Classifier Family** | **Capacity-Governing Hyperparameters Tuned** | **Effect of Increasing Complexity** |
| --- | --- | --- |
| **Logistic Regression** | *C*, penalty (L1/L2) | Higher *C* reduces bias but can increase variance. |
| **k-Nearest Neighbors** | *n_neighbors* | Lower *k* reduces bias but increases variance. |
| **Discriminate Analysis** | *shrinkage* (LDA), *reg_param* (QDA) | Less shrinkage/regularization reduces bias. |
| **Ensemble Trees** (RF, ET, GBC, XGB, LGBM) | *n_estimators*, *max_depth*, *num_leaves*, *learning_rate* | More estimators/deeper trees/higher learning rate can reduce bias but increase variance. |

**Table S4. Train–Test Gap (numeric)**

*Complete per-model values for train–test gap under LOPOCV and Nested LOPOCV, including variability (SD)*

| **Model** | **Train-Test Gap (LOPOCV ± SD)** | **Train-Test Gap (Nested CV ± SD)** |
| --- | --- | --- |
| **KNN** | 0.30 ± 0.03 | 0.30 ± 0.03 |
| **LR** | 0.08 ± 0.03 | 0.07 ± 0.03 |
| **ADA** | 0.25 ± 0.04 | 0.26 ± 0.04 |
| **LDA** | 0.08 ± 0.03 | 0.08 ± 0.03 |
| **QDA** | 0.13 ± 0.03 | 0.12 ± 0.04 |
| **GBC** | 0.34 ± 0.03 | 0.34 ± 0.03 |
| **ET** | 0.36 ± 0.04 | 0.35 ± 0.04 |
| **RF** | 0.36 ± 0.04 | 0.36 ± 0.04 |
| **XGB** | 0.35 ± 0.03 | 0.34 ± 0.03 |
| **LGBM** | 0.31 ± 0.03 | 0.34 ± 0.03 |

**Note**: Gap = Train Accuracy – Test Accuracy for each outer fold; averaged across participants.
LOPOCV = Leave-One-Participant-Out Cross-Validation; SD = Standard Deviation; CV = Cross-Validation. See Figures 3 for visualization of central tendency and spread.

**Table S5. Full evaluation metrics across models and validation strategies.**

| Model | Metric | 10-Fold CV | Group CV | LOPOCV | Nested CV |
| --- | --- | --- | --- | --- | --- |
| ADA | Accuracy | 0.77 ± 0.05 | 0.61 ± 0.34 | 0.63 ± 0.34 | 0.61 ± 0.34 |
|  | F1 Score | 0.76 ± 0.06 | 0.48 ± 0.44 | 0.48 ± 0.45 | 0.48 ± 0.44 |
|  | MCC | 0.54 ± 0.11 | 0.00 ± 0.00 | 0.00 ± 0.00 | 0.00 ± 0.00 |
|  | Precision | 0.76 ± 0.04 | 0.62 ± 0.49 | 0.61 ± 0.49 | 0.62 ± 0.49 |
|  | Recall | 0.76 ± 0.09 | 0.82 ± 0.29 | 0.83 ± 0.29 | 0.82 ± 0.29 |
| ET | Accuracy | 0.91 ± 0.03 | 0.66 ± 0.33 | 0.67 ± 0.34 | 0.66 ± 0.33 |
|  | F1 Score | 0.90 ± 0.03 | 0.51 ± 0.46 | 0.54 ± 0.45 | 0.51 ± 0.46 |
|  | MCC | 0.82 ± 0.05 | 0.00 ± 0.00 | 0.00 ± 0.00 | 0.00 ± 0.00 |
|  | Precision | 0.91 ± 0.05 | 0.65 ± 0.48 | 0.68 ± 0.47 | 0.65 ± 0.48 |
|  | Recall | 0.90 ± 0.05 | 0.83 ± 0.31 | 0.82 ± 0.31 | 0.83 ± 0.31 |
| GBC | Accuracy | 0.85 ± 0.03 | 0.66 ± 0.32 | 0.67 ± 0.31 | 0.66 ± 0.32 |
|  | F1 Score | 0.84 ± 0.04 | 0.48 ± 0.45 | 0.47 ± 0.45 | 0.48 ± 0.45 |
|  | MCC | 0.70 ± 0.07 | 0.00 ± 0.00 | 0.00 ± 0.00 | 0.00 ± 0.00 |
|  | Precision | 0.84 ± 0.04 | 0.61 ± 0.49 | 0.60 ± 0.49 | 0.61 ± 0.49 |
|  | Recall | 0.84 ± 0.06 | 0.83 ± 0.29 | 0.84 ± 0.29 | 0.83 ± 0.29 |
| KNN | Accuracy | 0.91 ± 0.04 | 0.68 ± 0.30 | 0.69 ± 0.29 | 0.68 ± 0.30 |
|  | F1 Score | 0.91 ± 0.04 | 0.51 ± 0.44 | 0.51 ± 0.45 | 0.51 ± 0.44 |
|  | MCC | 0.82 ± 0.08 | 0.00 ± 0.00 | 0.00 ± 0.00 | 0.00 ± 0.00 |
|  | Precision | 0.90 ± 0.06 | 0.61 ± 0.49 | 0.61 ± 0.49 | 0.61 ± 0.49 |
|  | Recall | 0.92 ± 0.03 | 0.85 ± 0.25 | 0.86 ± 0.25 | 0.85 ± 0.25 |
| LDA | Accuracy | 0.70 ± 0.06 | 0.63 ± 0.35 | 0.63 ± 0.35 | 0.63 ± 0.35 |
|  | F1 Score | 0.68 ± 0.06 | 0.48 ± 0.45 | 0.48 ± 0.45 | 0.48 ± 0.45 |
|  | MCC | 0.41 ± 0.12 | 0.00 ± 0.00 | 0.00 ± 0.00 | 0.00 ± 0.00 |
|  | Precision | 0.72 ± 0.07 | 0.64 ± 0.48 | 0.64 ± 0.48 | 0.64 ± 0.48 |
|  | Recall | 0.65 ± 0.08 | 0.80 ± 0.32 | 0.80 ± 0.32 | 0.80 ± 0.32 |
| LGBM | Accuracy | 0.79 ± 0.06 | 0.62 ± 0.33 | 0.62 ± 0.33 | 0.62 ± 0.33 |
|  | F1 Score | 0.79 ± 0.07 | 0.43 ± 0.44 | 0.43 ± 0.44 | 0.43 ± 0.44 |
|  | MCC | 0.59 ± 0.12 | 0.00 ± 0.00 | 0.00 ± 0.00 | 0.00 ± 0.00 |
|  | Precision | 0.78 ± 0.06 | 0.57 ± 0.50 | 0.56 ± 0.50 | 0.57 ± 0.50 |
|  | Recall | 0.79 ± 0.10 | 0.82 ± 0.31 | 0.83 ± 0.29 | 0.82 ± 0.31 |
| LR | Accuracy | 0.70 ± 0.06 | 0.64 ± 0.35 | 0.64 ± 0.35 | 0.64 ± 0.35 |
|  | F1 Score | 0.68 ± 0.07 | 0.50 ± 0.45 | 0.49 ± 0.45 | 0.50 ± 0.45 |
|  | MCC | 0.41 ± 0.13 | 0.00 ± 0.00 | 0.00 ± 0.00 | 0.00 ± 0.00 |
|  | Precision | 0.71 ± 0.07 | 0.65 ± 0.48 | 0.64 ± 0.48 | 0.65 ± 0.48 |
|  | Recall | 0.67 ± 0.09 | 0.82 ± 0.31 | 0.81 ± 0.31 | 0.82 ± 0.31 |
| QDA | Accuracy | 0.77 ± 0.05 | 0.66 ± 0.35 | 0.66 ± 0.34 | 0.66 ± 0.35 |
|  | F1 Score | 0.74 ± 0.06 | 0.52 ± 0.45 | 0.51 ± 0.45 | 0.52 ± 0.45 |
|  | MCC | 0.54 ± 0.11 | 0.00 ± 0.00 | 0.00 ± 0.00 | 0.00 ± 0.00 |
|  | Precision | 0.80 ± 0.06 | 0.68 ± 0.47 | 0.67 ± 0.47 | 0.68 ± 0.47 |
|  | Recall | 0.69 ± 0.09 | 0.80 ± 0.32 | 0.80 ± 0.32 | 0.80 ± 0.32 |
| RF | Accuracy | 0.85 ± 0.02 | 0.65 ± 0.34 | 0.64 ± 0.34 | 0.65 ± 0.34 |
|  | F1 Score | 0.84 ± 0.03 | 0.50 ± 0.45 | 0.50 ± 0.45 | 0.50 ± 0.45 |
|  | MCC | 0.71 ± 0.05 | 0.00 ± 0.00 | 0.00 ± 0.00 | 0.00 ± 0.00 |
|  | Precision | 0.86 ± 0.02 | 0.64 ± 0.48 | 0.65 ± 0.48 | 0.64 ± 0.48 |
|  | Recall | 0.83 ± 0.07 | 0.82 ± 0.31 | 0.81 ± 0.32 | 0.82 ± 0.31 |
| XGB | Accuracy | 0.87 ± 0.05 | 0.66 ± 0.33 | 0.64 ± 0.33 | 0.66 ± 0.33 |
|  | F1 Score | 0.86 ± 0.05 | 0.48 ± 0.45 | 0.46 ± 0.44 | 0.48 ± 0.45 |
|  | MCC | 0.73 ± 0.10 | 0.00 ± 0.00 | 0.00 ± 0.00 | 0.00 ± 0.00 |
|  | Precision | 0.87 ± 0.06 | 0.61 ± 0.49 | 0.60 ± 0.49 | 0.61 ± 0.49 |
|  | Recall | 0.85 ± 0.07 | 0.83 ± 0.29 | 0.82 ± 0.30 | 0.83 ± 0.29 |

Complete per-model results (Accuracy, F1, Precision, Recall, MCC) under 10-Fold CV (trial-wise), Group K-Fold (K=3) (subject-aware), LOPOCV, and Nested LOPOCV + Group K-Fold (K=3). Values are reported as mean ± SD and align with the summary trends in Table 3 and Section 3.1. Use this table for model-by-model comparisons beyond accuracy.

**Table S6. Full bias matrix across validation strategies and metrics.**

| Model | Baseline | Compared To | | Accuracy Δ | F1 Score Δ | Precision Δ | Recall Δ | MCC Δ | MAB |
| --- | --- | --- | --- | --- | --- | --- | --- | --- | --- |
| KNN | LOPOCV | | Group CV | -0.003 | -0.004 | 0 | -0.006 | 0 | 0.003 |
|  | LOPOCV | | 10-Fold CV | 0.219 | 0.397 | 0.285 | 0.059 | 0.816 | 0.355 |
|  | Nested LOPOCV | | Group CV | 0 | 0 | 0 | 0 | 0 | 0 |
|  | Nested LOPOCV | | 10-Fold CV | 0.223 | 0.401 | 0.285 | 0.065 | 0.816 | 0.358 |
| LR | LOPOCV | | Group CV | 0.003 | 0.016 | 0.014 | 0.003 | 0 | 0.007 |
|  | LOPOCV | | 10-Fold CV | 0.064 | 0.195 | 0.066 | -0.145 | 0.407 | 0.175 |
|  | Nested LOPOCV | | Group CV | 0 | 0 | 0 | 0 | 0 | 0 |
|  | Nested LOPOCV | | 10-Fold CV | 0.061 | 0.18 | 0.052 | -0.148 | 0.407 | 0.17 |
| AB | LOPOCV | | Group CV | -0.014 | 0.006 | 0.014 | -0.01 | 0 | 0.009 |
|  | LOPOCV | | 10-Fold CV | 0.142 | 0.282 | 0.151 | -0.066 | 0.542 | 0.237 |
|  | Nested LOPOCV | | Group CV | 0 | 0 | 0 | 0 | 0 | 0 |
|  | Nested LOPOCV | | 10-Fold CV | 0.156 | 0.276 | 0.137 | -0.056 | 0.542 | 0.233 |
| LDA | LOPOCV | | Group CV | 0 | 0 | 0 | 0 | 0 | 0 |
|  | LOPOCV | | 10-Fold CV | 0.072 | 0.2 | 0.077 | -0.152 | 0.411 | 0.182 |
|  | Nested LOPOCV | | Group CV | 0 | 0 | 0 | 0 | 0 | 0 |
|  | Nested LOPOCV | | 10-Fold CV | 0.072 | 0.2 | 0.077 | -0.152 | 0.411 | 0.182 |
| QDA | LOPOCV | | Group CV | -0.002 | 0.014 | 0.014 | 0.002 | 0 | 0.006 |
|  | LOPOCV | | 10-Fold CV | 0.104 | 0.234 | 0.133 | -0.109 | 0.539 | 0.224 |
|  | Nested LOPOCV | | Group CV | 0 | 0 | 0 | 0 | 0 | 0 |
|  | Nested LOPOCV | | 10-Fold CV | 0.107 | 0.22 | 0.119 | -0.11 | 0.539 | 0.219 |
| GBC | LOPOCV | | Group CV | -0.014 | 0.007 | 0.014 | -0.008 | 0 | 0.009 |
|  | LOPOCV | | 10-Fold CV | 0.175 | 0.368 | 0.248 | 0.003 | 0.697 | 0.298 |
|  | Nested LOPOCV | | Group CV | 0 | 0 | 0 | 0 | 0 | 0 |
|  | Nested LOPOCV | | 10-Fold CV | 0.189 | 0.361 | 0.234 | 0.011 | 0.697 | 0.298 |
| ET | LOPOCV | | Group CV | -0.01 | -0.025 | -0.028 | 0.004 | 0 | 0.013 |
|  | LOPOCV | | 10-Fold CV | 0.243 | 0.368 | 0.234 | 0.076 | 0.819 | 0.348 |
|  | Nested LOPOCV | | Group CV | 0 | 0 | 0 | 0 | 0 | 0 |
|  | Nested LOPOCV | | 10-Fold CV | 0.253 | 0.393 | 0.262 | 0.072 | 0.819 | 0.36 |
| RF | LOPOCV | | Group CV | 0.005 | -0.007 | -0.014 | 0.007 | 0 | 0.007 |
|  | LOPOCV | | 10-Fold CV | 0.208 | 0.341 | 0.208 | 0.017 | 0.707 | 0.296 |
|  | Nested LOPOCV | | Group CV | 0 | 0 | 0 | 0 | 0 | 0 |
|  | Nested LOPOCV | | 10-Fold CV | 0.203 | 0.347 | 0.221 | 0.01 | 0.707 | 0.298 |
| XGB | LOPOCV | | Group CV | 0.021 | 0.02 | 0.014 | 0.008 | 0 | 0.013 |
|  | LOPOCV | | 10-Fold CV | 0.224 | 0.4 | 0.271 | 0.031 | 0.733 | 0.332 |
|  | Nested LOPOCV | | Group CV | 0 | 0 | 0 | 0 | 0 | 0 |
|  | Nested LOPOCV | | 10-Fold CV | 0.203 | 0.38 | 0.257 | 0.023 | 0.733 | 0.319 |
| LGBM | LOPOCV | | Group CV | 0.005 | 0.002 | 0.014 | -0.012 | 0 | 0.007 |
|  | LOPOCV | | 10-Fold CV | 0.176 | 0.36 | 0.227 | -0.039 | 0.59 | 0.278 |
|  | Nested LOPOCV | | Group CV | 0 | 0 | 0 | 0 | 0 | 0 |
|  | Nested LOPOCV | | 10-Fold CV | 0.171 | 0.358 | 0.213 | -0.027 | 0.59 | 0.272 |

Pairwise bias (Δ) of each strategy versus a subject-aware baseline (LOPOCV or Nested CV) for all models and all metrics (Accuracy, F1, Precision, Recall, MCC), including Mean Absolute Bias (MAB). This expands the illustrative subset shown in Table 5 and supports claims in Section 3.3 about inflation under trial-wise CV and the close agreement among subject-aware schemes.

**Figure S1. Heatmap of the full bias matrix.**
*Visualization of Table S6: color intensity encodes the magnitude of absolute bias per model × metric × strategy comparison. Warmer cells indicate larger overestimation relative to the subject-aware baseline; cooler cells indicate small or zero bias. This figure provides a quick, pattern-level view that complements the numeric matrix and supports Section 3.3.*


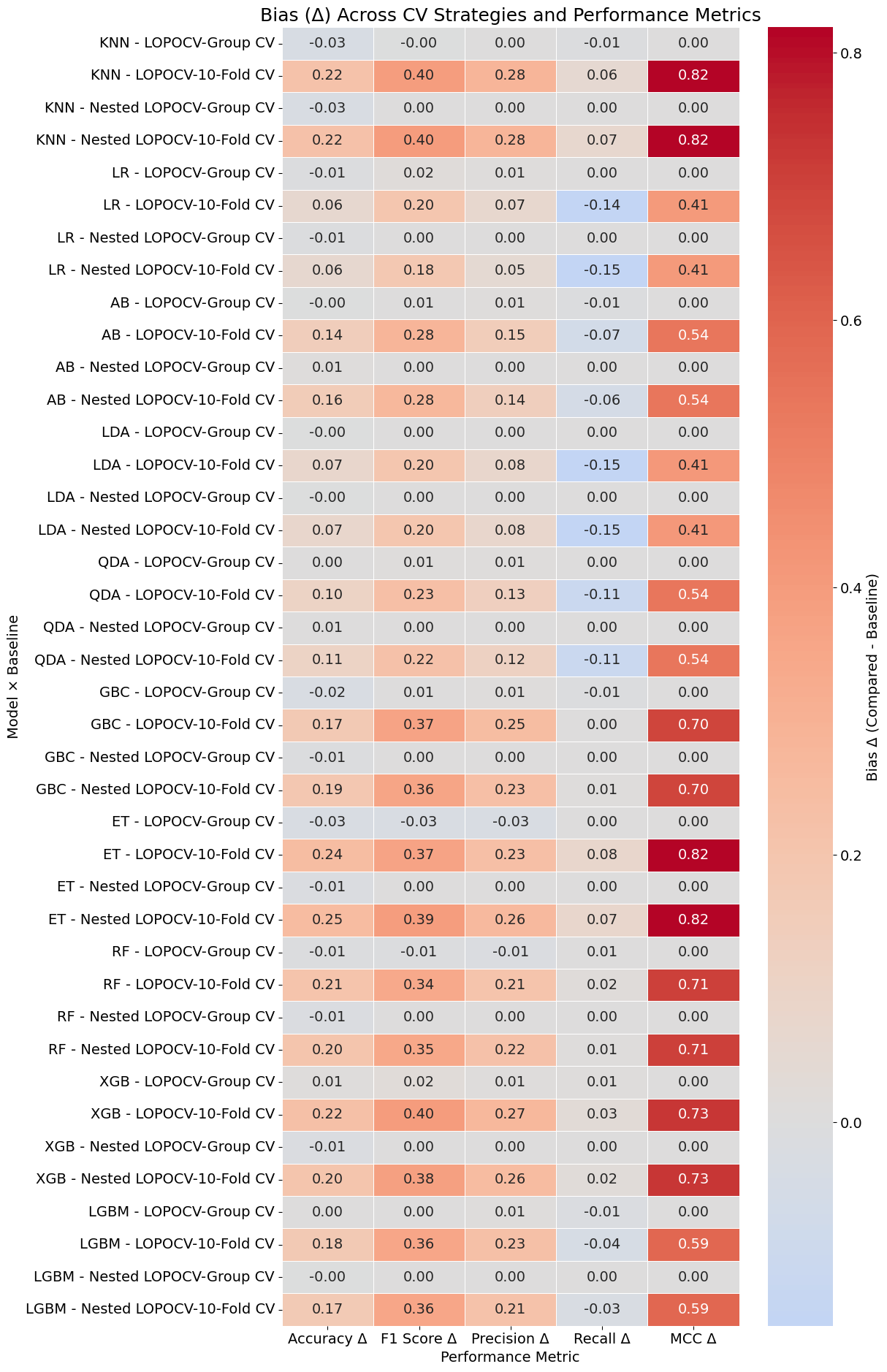


**Appendix 3— Cross-Validation Algorithms (procedural reference)**

This section provides the procedural recipes used to evaluate models on repeated-measures data. We distinguish trial-wise validation from subject-aware validation. Trial-wise splits may place correlated trials from the same participant in both training and test sets, which can inflate performance; subject-aware splits keep all trials from a participant in a single fold. Preprocessing (e.g., standardization) is fit on training data only and then applied to validation/test data within each fold. Metrics are averaged across folds, and when applicable we report the train–test gap computed on the outer folds. The algorithm boxes below include all Inputs/Outputs and step-wise procedures. For reproducibility, use a fixed random seed where shuffling applies and maintain a group vector aligned with trials.

**Algorithm S1.** Standard k-Fold Cross-Validation (trial-wise; non-subject-aware ).

| ***Input:*** *Full dataset* $D$*, model* $M$ *with fixed hyperparameters, number of folds* $k$ | |
| --- | --- |
| ***Output:*** *Aggregated evaluation metrics* $M_{agg}$ |  |
| 1. Split $D$ into $k$ stratified folds $F_{1},F_{2},...,F_{k}$ | ▷ Random trial-wise partition |
| 2. **for** each fold $j\in\{1,...,k\}$ **do** | ▷ Iterate through folds |
| 3.      $D_{test}\leftarrow F_{j}$;   $D_{train}\leftarrow D\backslash D_{test}$ | ▷ Assign training and test sets |
| 4.      Standardize($D_{train},D_{test}$) | ▷ Fit on train, apply to both |
| 5.      model $\leftarrow$ Train($M,D_{train}$) | ▷ Train on the training data |
| 6.      $M_{j}\leftarrow$ Evaluate(model, $D_{test}$) | ▷ Compute metrics for current fold |
| 7. **end for** |  |
| 8. $M_{agg}\leftarrow$ aggregate({$M_{j}$}) | ▷ Average metrics across all folds |
| 9. **return** $M_{agg}$ |  |

Baseline trial-level validation that preserves class balance and serves as a comparator.

**Algorithm S2.** Leave-One-Participant-Out Cross-Validation (LOPOCV) (subject-aware ; fixed hyperparameters).

| ***Input:*** *Full dataset* $D$*, participant set* $P$*, model* $M$ *with fixed hyperparameters* | |
| --- | --- |
| ***Output:*** *Aggregated metrics* $M_{agg}$*, aggregated Train-Test Gap* $G_{agg}$ | |
| 1. **for** each participant $p\in P$ **do** | ▷ LOPOCV fold |
| 2.      $D_{test}\leftarrow\{p\}$;   $D_{train}\leftarrow D\backslash\{p\}$ | ▷ Test on one participant, train on all others |
| 3.      Standardize($D_{train},D_{test}$) | ▷ Fit on train, apply to both |
| 4.      model $\leftarrow$ Train($M,D_{train}$) | ▷ Train the model |
| 5.      ($M_{p},G_{p}$) $\leftarrow$ Evaluate(model, $D_{train},D_{test}$) | ▷ Compute metrics and Train-Test Gap |
| 6. **end for** |  |
| 7. $M_{agg}\leftarrow$ aggregate({$M_{p}$}) | ▷ Average metrics across participants |
| 8. $G_{agg}\leftarrow$ aggregate({$G_{p}$}) | ▷ Average gap across participants |
| 9. **return** $M_{agg},G_{agg}$ |  |

Evaluation on unseen individuals by holding out one participant per fold

**Algorithm S3.** Group K-Fold (K=3) Cross-Validation (subject-aware ).

| ***Input:*** *Dataset* $D$*, participant set* $P$*, model* $M$ *with fixed hyperparameters, number of groups* $k$ | |
| --- | --- |
| ***Output:*** *Aggregated evaluation metrics* $M_{agg}$ | |
| 1. Split $P$ into $k$ disjoint groups $G_{1},G_{2},...,G_{k}$ | ▷ Participant-wise partition |
| 2. **for** each group $j\in\{1,...,k\}$ **do** | ▷ Iterate through participant groups |
| 3.      $D_{test}\leftarrow$ trials from participants in $G_{j}$ | ▷ Construct test set |
| 4.      $D_{train}\leftarrow D\backslash D_{test}$ | ▷ Training set is all other participants |
| 5.      Standardize($D_{train},D_{test}$) | ▷ Fit on train, apply to both |
| 6.      model $\leftarrow$ Train($M,D_{train}$) | ▷ Train on the training data |
| 7.      $M_{j}\leftarrow$ Evaluate(model, $D_{test}$) | ▷ Compute metrics for current fold |
| 8. **end for** |  |
| 9. $M_{agg}\leftarrow$ aggregate({$M_{j}$}) | ▷ Average metrics across folds |
| 10. **return** $M_{agg}$ |  |

Participant-wise folds with multiple participants per test set; trades granularity for efficiency.

**Algorithm S4.** Nested CV: LOPOCV (outer) + Group K-Fold CV (K=3) (inner).

| ***Input:*** *Dataset* $D$*, participant set* $P$*, model class* $M_{class}$*, hyperparameter grid* $\Theta$ | |
| --- | --- |
| ***Output:*** *Aggregated unbiased metrics* $M_{agg}$*, aggregated Train-Test Gap* $G_{agg}$ | |
| 1. **Outer Loop for Unbiased Evaluation** |  |
| 2. **for** each participant $p_{outer}\in P$ **do** | ▷ LOPOCV outer fold |
| 3.      $D_{outer\_test}\leftarrow\{p_{outer}\}$;   $D_{outer\_train}\leftarrow D\backslash D_{outer\_test}$ | ▷ Define outer train/test split |
| 4.       **Inner Loop for Hyperparameter Tuning** |  |
| 5.      $P_{inner}\leftarrow P\backslash\{p_{outer}\}$ | ▷ Use remaining participants for tuning |
| 6.      Split $P_{inner}$ into $k_{inner}$ groups for Group CV | ▷ Prepare for inner cross-validation |
| 7.      $\theta^{*}\leftarrow$ Tune($M_{class},\Theta,D_{outer\_train}$, using Group CV) | ▷ Find best hyperparameter set |
| 8.      **Final Evaluation for this Outer Fold** |  |
| 9.      Standardize($D_{outer\_train},D_{outer\_test}$) | ▷ Fit on the full outer training set |
| 10.     final_model $\leftarrow$ Train($M_{class}(\theta^{*}),D_{outer\_train}$) | ▷ Train final model with best params |
| 11.     ($M_{p},G_{p}$) $\leftarrow$ Evaluate(final_model, $D_{outer\_train},D_{outer\_test}$) | ▷ Evaluate on held-out participant |
| 12. **end for** |  |
| 13. $M_{agg}\leftarrow$ aggregate({$M_{p}$}) | ▷ Average final metrics across all participants |
| 14. $G_{agg}\leftarrow$ aggregate({$G_{p}$}) | ▷ Average final gaps across all participants |
| 15. **return** $M_{agg},G_{agg}$ |  |

Subject-aware nesting that decouples hyperparameter tuning from evaluation to avoid selection bias.
